# Comparison of *miRNA*-*101a*-*3p* and *miRNA*-*144a*-*3p* regulation with the key genes of alpaca melanocyte pigmentation

**DOI:** 10.1186/s12867-019-0137-8

**Published:** 2019-08-14

**Authors:** Zhiwei Zhu, Yueyue Ma, Yuan Li, Zhixue Cheng, Huifeng Li, Lihuan Zhang, Dongmei Xu, Pengfei Li

**Affiliations:** 1 College of Life Science, Shanxi Agricultural University, Taigu, 030801 China; 2 Department of Veterinary Pharmacology and Toxicology, College of Veterinary Medicine, China Agricultural University, Beijing, 100094 China; 3 College of Animal Science and Technology, Shanxi Agricultural University, Taigu, 030801 China

**Keywords:** Melanocyte, *miRNA*-*101a*-*3p*, *miRNA*-*144a*-*3p*, Melanin

## Abstract

**Background:**

Many miRNA functions have been revealed to date. Single miRNAs can participate in life processes by regulating more than one target gene, and more than one miRNA can also simultaneously act on one target mRNA. Thus, a complex regulatory network involved in many processes can be formed. Herein, the pigmentation regulation mechanism of *miR*-*101a*-*3p* and *miR*-*144a*-*3p* was studied at the cellular level by the overexpression and equal overexpression of *miR*-*101a*-*3p* and *miR*-*144a*-*3p.*

**Results:**

Results revealed that *miR*-*101a*-*3p* and *miR*-*144a*-*3p* directly regulated the expression of microphthalmia-associated transcription factor, thereby affecting melanin synthesis.

**Conclusions:**

The two miRNAs with the same binding site in the same gene independently excreted each other’s function. However, the inhibitory effect of *miR*-*144a*-*3p* was stronger than that of *miR*-*101*-*3p* in alpaca melanocytes, although both decreased melanin production.

## Background

As agricultural and companion animals, alpacas (*Vicugna pacos*) have a great variety of natural coat colors. The alpaca’s hair, known as soft gold, has considerable economic value, and it has become a good model for studying animal hair color genes [1]. Melanocytes are present in mammalian hair, skin, and irises. In these tissues, the distribution, proliferation, survival, and number and type of melanocytes, as well as the production of melanin, are regulated by various factors [2, 3]. At present, more than 400 genes have been found to regulate animal hair color. A series of genes can regulate melanocyte development and melanin [4, 5]. Microphthalmia-associated transcription factor (MITF) is an important regulator of melanocyte survival and development, and MITF controls their transcription by binding to tyrosinase (TYR) and TYR-related proteins 1 (TYRP1) and 2 (TYRP2) promoter regions, which are predominantly involved in melanin synthesis [6, 7]. In animals, miRNAs inhibit target mRNA translation by pairing with the specific bases of the target mRNA gene; miRNAs are involved in regulating gene expression and a range of biological functions, such as cell differentiation and tissue and tumor development [8, 9]. Numerous miRNAs have been discovered with the development of sequencing technology. Single miRNAs can participate in life processes by regulating more than one target gene, and more than one miRNA can also simultaneously act on one target mRNA [10, 11]. Thus, a complex regulatory network involved in many processes, such as skin tissue development, melanocyte formation, pigment cell migration, and melanin formation, is formed [12, 13].

In the present study, TargetScan software demonstrated that *miR*-*101*-*3p* and *miR*-*144*-*3p* exhibited the same binding site on the 3′UTR of *MITF*. The functions of *miR*-*101*-*3p* and *miR*-*144*-*3p* have been verified in many tumors, but no study on pigmentation in melanocytes has been conducted. Can *miR*-*101*-*3p* and *miR*-*144*-*3p* regulate hair color? Can both of them inhibit the same target gene?

## Materials and methods

### Cell culture

Alpaca melanocytes were established and maintained at the Alpaca Biological Engineering Laboratory (Shanxi Agricultural University, Taigu, Jinzhong, Shanxi. China) [14, 15]. Alpacas were for research animals from Alpaca Farm (Zhuangzi, Yuci, Jinzhong, Shanxi, China). Housing and care of the alpacas and collection of skin samples were approved by the Animal Experimentation Ethics Committee of Shanxi Agricultural University, Taigu, China (SXAU-EAW-2019-L013003). Punch skin biopsies (4 × 8 mm) were obtained from alpacas under local anesthesia [14]. After the skin tissues were taken, debridement therapy was performed for the alpacas’ injury. The sixth-passage alpaca melanocytes from the Alpaca Biological Engineering Laboratory were used in this experiment.

The frozen melanocytes were resuscitated in a water bath at 37 °C, and an equal amount of Dulbecco’s minimum essential medium (DMEM; Gibco, New York, NY, USA) with 10% fetal bovine serum was added and centrifuged at 1000*g*/min for 10 min. The supernatant was discarded, and the cell pellet was suspended in melanocyte medium (MelM; ScienCell, Carlsbad, CA, USA). The cells were seeded into six-well plates and maintained at 37 °C and 5% CO_2_. Some cells were used to isolate total RNA, and some were further cultured for transfection experiments.

HEK 293T cells were purchased from Ribobio (Guangzhou, China), seeded into six-well plates, and maintained (37 °C, 5% CO_2_) in DMEM (Gibco, New York, USA) supplemented with 10% fetal bovine serum. HEK 293T cells were prepared for luciferase assays.

### miR-101a-3p and miR-144a-3p bioinformatics analysis

The homology of *miR*-*101a*-*3p, miR*-*144a*-*3p*, and *MITF* 3′UTR in human, bovine, chicken, mouse, camel, and other species was analyzed using DNAMAN (LynnonBiosoft, USA). The target relationship among *miR*-*101a*-*3p, miR*-*144a*-*3p*, and *MITF* was predicted using TargetScan (TargetScan 5.2, https://www.targetscan.org).

### Luciferase assays

To examine the *MITF* 3′UTR as a target of *miR*-*101a*-*3p* and *miR*-*144a*-*3p* in vitro, luciferase assays were performed using a pmiRGLO Dual-Luciferase miRNA Target Expression Vector (Promega, Fitchburg, WI, USA), containing both the coding sequences of firefly luciferase and Renilla luciferase (internal control). The *miR*-*144a*-*3p* and *miR*-*101a*-*3p* binding site or mismatched site of *MITF* 3′UTR (NW 005882703.1) was cloned into the pmiRGLO vector system. HEK 293T cells were transfected (48 h) with either wild type (WT) or mutant pmirGLO dual-luciferase vector (100 ng) or miRNA mimics (50 nM) by using Lipofectamine 2000 (Invitrogen, Carlsbad, CA, USA) following the manufacturer’s instructions. A dual-luciferase reporter assay system (Promega) was then used to stimulate a luminescent signal that was measured by a luminometer (Glomax; Promega). Luminescence values were normalized to those produced by co-transfected Renilla luciferase constructs [15]. An empty pmiRGLO vector was used as a background control. Normalized firefly luciferase activity (firefly luciferase activity/Renilla luciferase activity) for each construct was compared with that of the empty pmiRGLO vector control. For each transfection, luciferase activity was averaged from three replicates.

### Transfection

*miR*-*101a*-*3p* and *miR*-*144a*-*3p* mimics and inhibitors were synthesized by Ribobio (Ribobio, Guangzhou, China). Melanocyte transfections were performed using riboFECT™ CP (Ribobio) in accordance with the manufacturer’s protocol. Approximately 5 μL of 20 μM miRNA mimic was diluted with 120 μL of 1× riboFECT™ CP buffer and added with 12 μL of riboFECT™ CP reagent. The mixture was incubated for 15 min at room temperature. The prepared riboFECT™ CP mixture was added to 1863 μL of cell culture medium. The melanocyte density reached 50% during transfection. The melanocytes were confluent in six-well plates. The culture plate was placed in a CO_2_ incubator at 37 °C for 48 h, and the cells were then collected.

### RNA isolation and reverse transcription

The melanocytes were confluent in the six-well plate. The medium in the culture plate was discarded, and the cells were gently washed three times with PBS (Ca^2+^-free, Mg^2+^-free) at 37 °C. PBS was then discarded. Total RNA was isolated from the cells by using a Trizol reagent (Invitrogen, Carlsbad, CA, USA). The concentration was assessed using a NanoDrop 2000c spectrophotometer (NanoDrop Technologies, Wilmington, DE, USA), and the quality was confirmed by 1% agarose gel electrophoresis. The isolated mRNAs and miRNAs were then reverse transcribed to cDNA using a SYBR Green I qRT-PCR (TaKaRa, Dalian, China) or miRcute miRNA SYBR Green I qRT-PCR kit (Tiangen, Beijing, China) in accordance with the manufacturer’s instructions, respectively.

### Quantitative real-time PCR detection of the expression of miR-101a-3p, miR-144a-3p, MITF, TYR, TYRP1, and TYRP2

The test was divided into seven groups: control (alpaca melanocytes), *miR*-*101a*-*3p* mimic (alpaca melanocytes transfected with *miR*-*101a*-*3p* mimic), *miR*-*144a*-*3p* mimic (alpaca melanocytes transfected with *miR*-*144a*-*3p* mimic), *miR*-*101a*-*3p* and *miR*-*144a*-*3p* mimic (alpaca melanocytes transfected with *miR*-*101a*-*3p and miR*-*144a*-*3p* mimic, 1:1), *miR*-*101a*-*3p* inhibitor (alpaca melanocytes transfected with *miR*-*101a*-*3p* inhibitor), *miR*-*144a*-*3p* inhibitor (alpaca melanocytes transfected with *miR*-*144a*-*3p* inhibitor), and *miR*-*101a*-*3p* and *miR*-*144a*-*3p* inhibitor groups (alpaca melanocytes transfected with *miR*-*101a*-*3p and miR*-*144a*-*3p* inhibitor, 1:1). Quantitative real-time PCR reaction system and reaction conditions were established following the instructions of the miRcute miRNA Fluorescence Quantification Kit (Tiangen, China). The forward primers for *miR*-*101a*-*3p*, *miR*-*144a*-*3p*, and *U6* snRNA are shown in Table 1. All primer sequences are listed in Table 1. The primers were designed by Primer3.0 plus according to these genes (*MITF*: XM_011241244, NW 005882703.1; *TYR*: NM_011661.5, MW 005882729.1; *TYRP1*: EU760771.1, NW 005882785.1; *TYRP2*: XM_006518510.3, NW 005882822.1; *18S rRNA*: NG032038.1. NW 006019996.1). The reaction system was as follows. The 10 µL PCR reaction included 5 µL of miRcute miRNA premix (with SYBR & ROX), 0.2 µL of forward primer (10 pM), 0.2 µL of universal qPCR primer (10 pM), 1 µL of template, and 3.6 µL of water. The reactions were incubated in a 96-well plate at 95 °C for 30 s, followed by 40 cycles at 95 °C for 5 s, 55 °C for 15 s, and 72 °C 20 s. After 55 °C for 30 s, the temperature was raised to 95 °C to form the melt curves. The abundance of *miR*-*101a*-*3p* and *miR*-*144a*-*3p* was normalized relative to that of *U6* snRNA. The abundance of target genes mRNA (*MITF, TYR, TYRP1,* and *TYRP2*) was normalized relative to that of *18S rRNA*. The produced transcripts were amplified using the ABI StepOnePlus Real-Time qPCR system (Thermo Fisher Scientific, Waltham, MA, USA), and all reactions were performed in triplicate [15].

**Table 1:** Primers used in this study

Gene	Primers (5′–3′)
*MITF*	
F	CGAAAGTTGCAACGRGAACAGCA
R	GAGCCT GCATTTCAAGTTCCTGTA
*TYR*	
F	TCTGGACCTCAGTTCCCCTTC
R	AACTTACAGTTTCCGCAGTTGA
*TYRP1*	
F	TGGCACAATGACGTATTCTTAGT
R	GGGTAGGAGGTAGGAGATGATG
*TYRP2*	
F	AGCAGACGGAACACTGGACT
R	GCATCTGTGGAAGGGTTGTT
18S	
F	GAAGGGCACCACCAGGAGT
R	CAGACAAATCACTCCA
*miR*-*101a*-*3p*	TACAGTACTGTGATAACTGAA
*miR*-*144a*-*3p*	TACAGTATAGATGATGTACT
U6	ATGGACTATCATATGCTTACCGTA

### Western blot analysis

Whole-cell protein was extracted from melanocytes using a protein extraction kit (Solarbio, Beijing, China), and the concentrations of the isolated cell lysates were determined spectrophotometrically by using a NanoDrop 2000c spectrophotometer (NanoDrop Technologies). The protein samples were added to 1% SDS and 5× protein loading buffer and boiled for 10 min for protein denaturation. Heat-denatured protein samples (200 ng per lane) were resolved via sodium dodecyl sulfate polyacrylamide gel electrophoresis and transferred to nitrocellulose membranes (Boster, Wuhan, China). The membranes were placed in blocking solution (5% non-fat dehydrated milk) and blocked at room temperature on a shaker (1 h, 80 r/min). The membranes were then placed in the diluted primary antibody at 4 °C overnight with mouse monoclonal MITF (Thermo Fisher Scientific) or rabbit monoclonal TYR, TYRP1, or TYRP2 (Abcam, Cambridge, UK) antibodies (1:2000 in Tris-buffered saline supplemented with Tween-20 [TBST]). A mouse monoclonal antibody to β-actin (1:3000 in TBST; TransGen Biotech, Beijing, China) was used as the control. The membranes were washed three times with TBST for 10 min each time. The membranes were placed in the diluted secondary antibody with horseradish peroxidase-conjugated goat anti-rabbit or anti-mouse secondary antibodies (1:1700 in TBST; Cwbio, Beijing, China), incubated at 37 °C for 1 h, and washed six times with TBST for 5 min each time. Finally, the membranes were detected by using a Superstar ECL Plus ready-to-use kit (Boster) according to the manufacturer’s instructions. The membranes were scanned on a ChemiDoc XRS + imager (Bio-Rad, Hercules, CA, USA), and the intensities of the generated protein signals were quantified using Image-Pro Plus software (Media Cybernetics, Inc., Georgia, MD, USA). The methodology has been published previously, because those protein detection conditions were same [15].

### Immunohistochemical analysis

The glass slides were placed in a 24-well culture plate. The frozen melanocytes were resuscitated in a 37 °C water bath, and an equal amount of DMEM and 10% fetal bovine serum was added. The mixture was centrifuged at 1000*g*/min for 10 min, and the supernatant was discarded. The cell pellet was suspended in melanocyte culture medium, dispensed in a 24-well plate, and maintained at a cell incubator (37 °C, 5% CO_2_).

The cells on the glass slides were fixed with 4% paraformaldehyde (4 °C, 30 min), incubated with 3% hydrogen peroxide (25 °C, 20 min), and blocked with blocking solution (37 °C, 35 min). The primary antibody (1:100) was added and reacted at 4 °C overnight, and the secondary antibody (1:200) was added and reacted at 37 °C for 30 min. The cells were stained with DAB and hematoxylin for 6 min and 30 s, respectively. The cells were then dehydrated with 70%, 80%, 90%, 95% ethanol; 100% ethanol I, and 100% ethanol II for 2, 2, 4, 4, 4 and 4 min, respectively. The cells were made transparent by using xylene I and II for 10 and 10 min, respectively. Finally, the glass slides were sealed with neutral resin.

### Melanin determination

Melanocytes were collected, washed three times with PBS, and subjected to cell counting. The cells were lysed using 0.2 mol/L NaOH (10^6^ cells/mL) and dispensed in a 96-well plate. The absorbance of the samples was measured at a wavelength of 475 nm [16, 17] by using a Multiskan Spectrum microplate reader (Thermo Fisher Scientific). Each group was repeated three times.

### Statistical analyses

The results of real-time quantitative PCR were quantified using the comparative threshold cycle method established by Livak and Schmittgen [18], and gene expression levels were normalized to those of *U6* and *18S rRNA*. Immunohistochemistry was used to analyze the optical density by using Image pro plus. Data were presented as the mean ± standard error. Significant (*P* < 0.05 or *P* < 0.01) or non-significant (*P* > 0.05) differences were evaluated via ANOVA using SPSS (SPSS, Inc., Chicago, IL, USA) and GraphPad Prism software (MacStats, UCI Graduate School of Management).

## Result

### Bioinformatics analysis of miR-101a-3p and miR-144a-3p

**Fig. 1:**
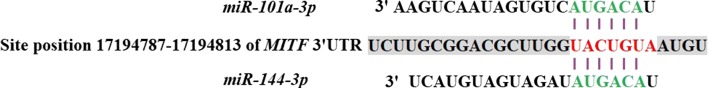
*miR*-*101a*-*3p* and *miR*-*144a*-*3p* binding sites of *MITF* 3′UTR. Red bases are the binding sites of *miR*-*101a*-*3p* and *miR*-*144a*-*3p* to *MITF* of *MITF* 3′UTR

*MITF* is a putative target gene of *miR*-*101a*-*3p* and *miR*-*144a*-*3p* was predicted by TargetScan. The *miR*-*101a*-*3p* and *miR*-*144a*-*3p* binding sites of *MITF* 3′UTR were the same (Fig. 1).

### Confirmation of the MITF 3′UTR as a target of miR-101a-3p and miR-144a-3p by luciferase reporter assays

**Fig. 2:**
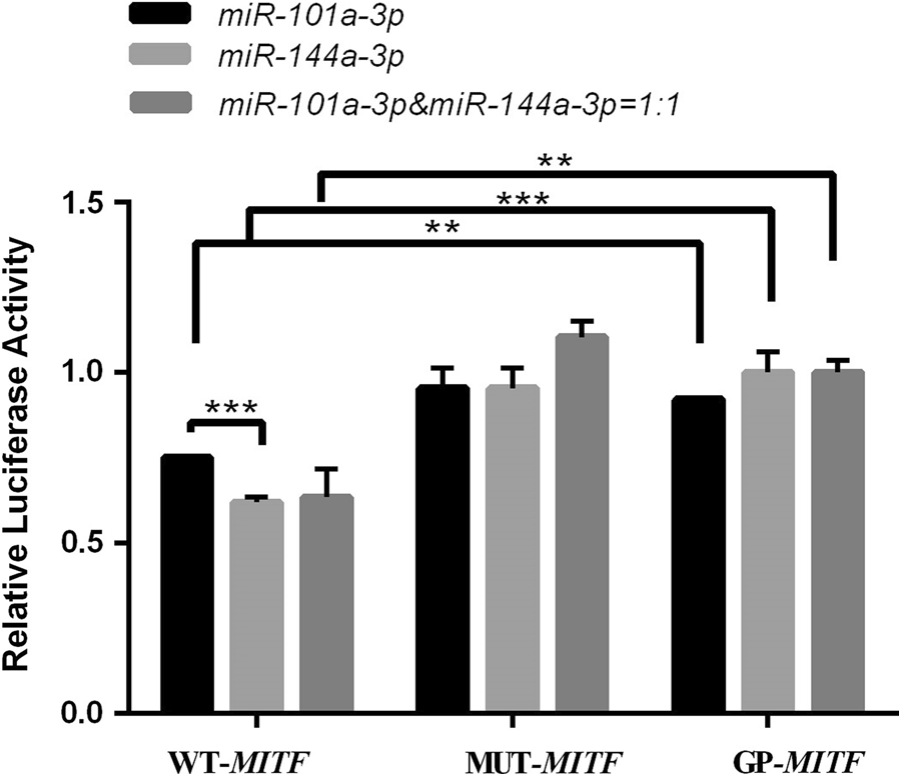
Effect of *miR*-*101a*-*3p* and *miR*-*144a*-*3p* on microphthalmia-associated transcription factor (MITF) expression. HEK 293T cells were co-transfected with luciferase reporter plasmids carrying *MITF* 3′UTR sequences that were wild type (WT-*MITF*), mutated at miR-148a-3p binding site (MUT-*MITF*), or empty vector (GP-*MITF*). The luciferase activity of each sample was normalized to Renilla luciferase activity. Bars in each panel represent the mean ± standard error. **P < 0.01; ***P < 0.001

Co-transfection of *miR*-*101a*-*3p* and *miR*-*144a*-*3p* mimics and the *MITF* 3′UTR target sequence into HEK 293T resulted in a decrease in luciferase activity compared with the empty vector control (*P *< 0.01 and *P *< 0.001, respectively; Fig. 2). Moreover, *miR*-*144a*-*3p* was stronger than *miR*-*101a*-*3p* (*P *< 0.001; Fig. 2). By contrast, luciferase activity was not decreased by co-transfection of the *MITF* 3′UTR target sequence carrying the *miR*-*101a*-*3p* and *miR*-*144a*-*3p* binding sites in the mutated form compared with the empty construct (Fig. 2), thereby indicating the interaction of *miR*-*101a*-*3p* and *miR*-*144a*-*3p* with the predicted binding sites in the *MITF* 3′UTR.

### miR-101a-3p and miR-144a-3p-modulated MITF and its downstream gene expression

**Fig. 3:**
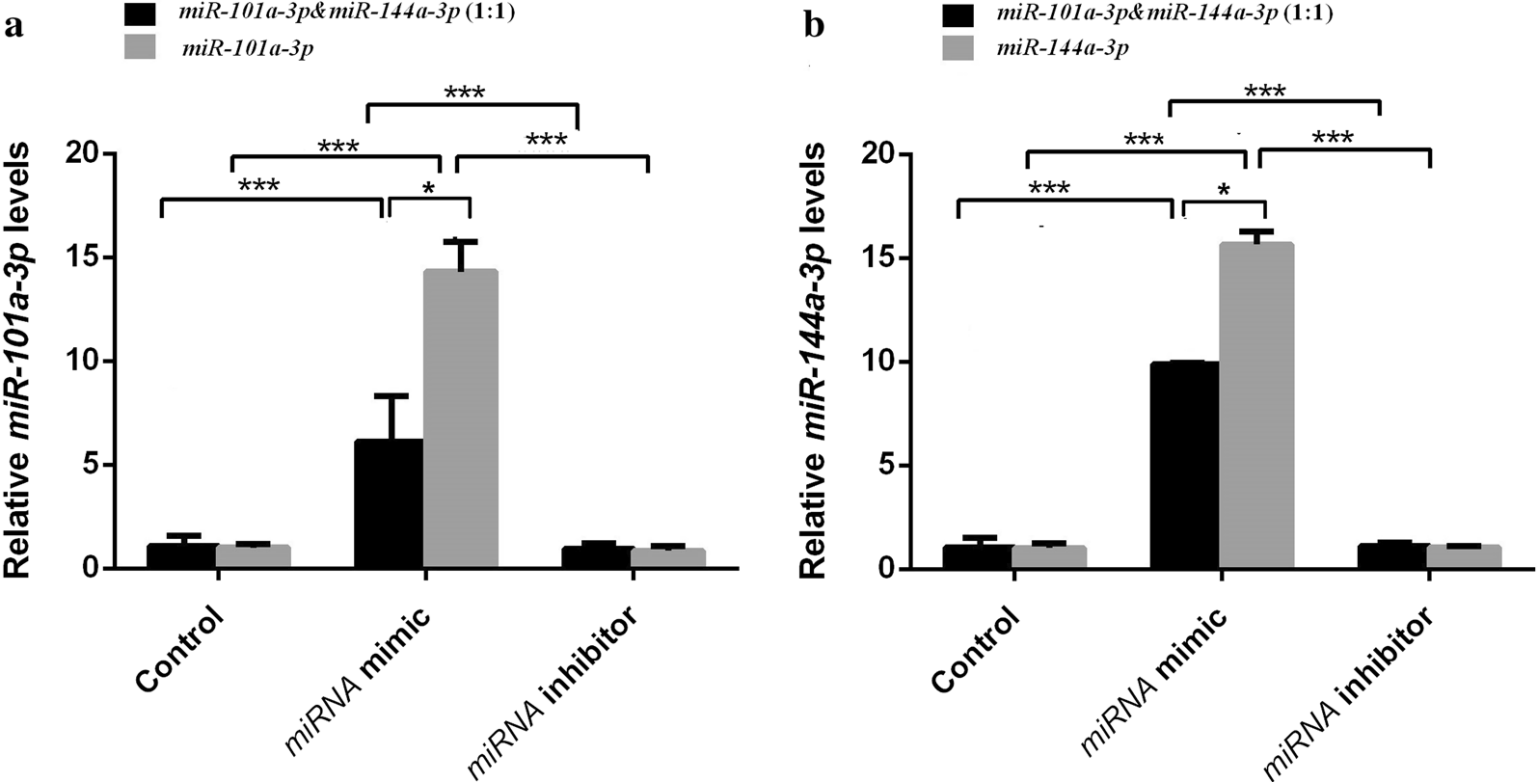
Expression of *miR*-*101a*-*3p* and *miR*-*144a*-*3p* in alpaca melanocytes. **a**, **b** represent the relative expression of *miR*-*101a*-*3p* and *miR*-*144a*-*3p*, respectively. The control group represents normal cultured melanocytes. miRNA mimic group represents melanocytes transfected with miRNA mimic. The black column represents the alpaca melanocytes transfected with *miR*-*101a*-*3p* & *miR*-*144a*-*3p* (1:1) mimic. The light gray column represents the alpaca melanocytes transfected with *miR*-*101a*-*3p* and *miR*-*144a*-*3p* mimics in **a** and **b**, respectively. The miRNA inhibitor group represents melanocytes transfected with miRNA inhibitor. The black column represents the alpaca melanocytes transfected with *miR*-*101a*-*3p* & *miR*-*144a*-*3p* (1:1) inhibitor, and the light gray column represents the alpaca melanocytes transfected with *miR*-*101a*-*3p* and *miR*-*144a*-*3p* inhibitors in **a** and **b**, respectively. (* means *P *< 0.05, ** means *P *< 0.01, *** means *P *< 0.001)

In the melanocytes transfected with *miR*-*144a*-*3p* mimic, *miR*-*101a*-*3p* mimic, and *miR*-*101a*-*3p* & *miR*-*144a*-*3p* (1:1) mimic groups, the expression level of *miR*-*144a*-*3p* or *miR*-*101a*-*3p* was significantly higher than that of the control groups (*P *< 0.001; Fig. 3). In the melanocytes transfected with *miR*-*144a*-*3p* mimic or *miR*-*101a*-*3p* mimic groups, the expression level of *miR*-*144a*-*3p* or *miR*-*101a*-*3p* was significantly higher than that of the transfected *miR*-*101a*-*3p* & *miR*-*144a*-*3p* (1:1) mimic group (*P *< 0.05; Fig. 3). The expression level of *miR*-*144a*-*3p* or *miR*-*101a*-*3p* in the melanocytes transfected with *miR*-*144a*-*3p*, *miR*-*101a*-*3p*, or *miR*-*101a*-*3p* & *miR*-*144a*-*3p* (1:1) inhibitors was significantly lower than that of the transfected *miR*-*144a*-*3p*, *miR*-*101a*-*3p*, *and miR*-*101a*-*3p* & *miR*-*144a*-*3p* (1:1) mimic groups (*P *< 0.001; Fig. 3).

**Fig. 4:**
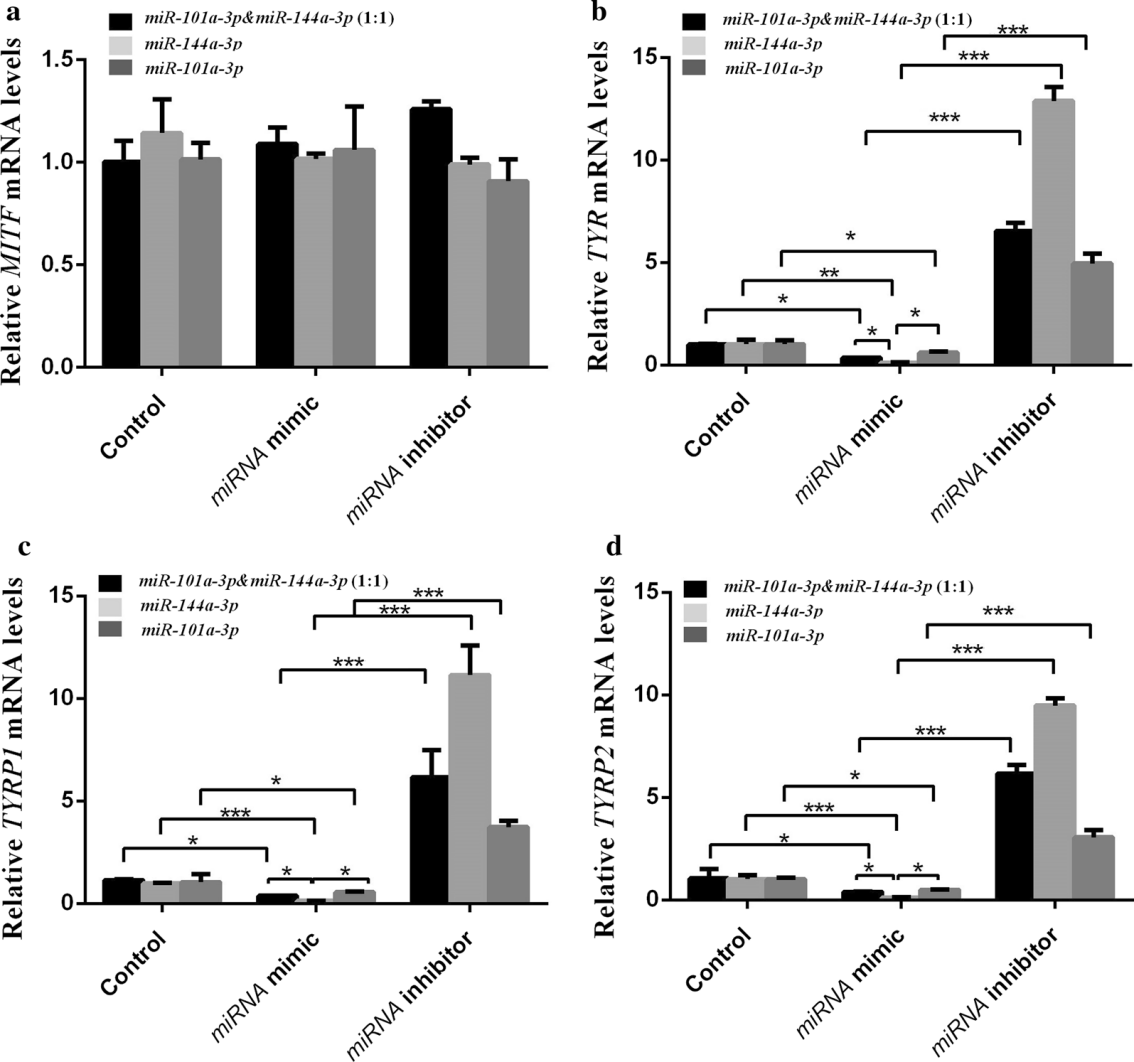
mRNA expression of *MITF*, *TYR*, *TYRP1*, and *TYRP2* in alpaca melanocytes. **a**–**d** represent the expression of MITF, TYR, TYRP1, and TYRP2, respectively. The control group represents normal cultured melanocytes. The miRNA mimic group represents melanocytes transfected with miRNA mimic. The black column represents the alpaca melanocytes transfected with *miR*-*101a*-*3p* & *miR*-*144a*-*3p* (1:1) mimic. The light gray column represents the alpaca melanocytes transfected with *miR*-*144a*-*3p* mimic. The dark gray column represents the alpaca melanocytes transfected with *miR*-*101a*-*3p* mimic. The miRNA inhibitor group represents melanocytes transfected with miRNA inhibitor. The black column represents the alpaca melanocytes transfected with *miR*-*101a*-*3p* & *miR*-*144a*-*3p* (1:1) inhibitor. The light gray column represents the alpaca melanocytes transfected with *miR*-*144a*-*3p* and *miR*-*101a*-*3p* inhibitors (* means *P *< 0.05, ** means *P* < 0.01, *** means *P* < 0.001)

The expression levels of *MITF* mRNA were not significant in the transfected miRNA mimic and inhibitor groups (Fig. 4a). The expression level of *TYR*, *TYRP1*, and *TYRP2* mRNA in the transfected *miR*-*144a*-*3p* mimic groups was significantly lower than that of the control group (*P *< 0.01, *P *< 0.001, and *P* < 0.001; Fig. 4b–d). The expression levels of *TYR, TYRP1*, and *TYRP2* mRNA in the transfected *miR*-*101a*-*3p* & *miR*-*144a*-*3p* (1:1) groups were lower than those of the control group (*P *< 0.05; Fig. 4b–d). However, the degree of inhibition was significantly less than that of the transfected *miR*-*144a*-*3p* mimic group (*P *< 0.05; Fig. 4b–d) but stronger than that of the *miR*-*101a*-*3p* mimic group (*P *> 0.05; Fig. 4b–d). The degree of *miR*-*144a*-*3p* inhibition to *TYR, TYRP1,* and *TYRP2* was significantly higher than that of transfected *miR*-*101a*-*3p* mimic group (*P *< 0.05; Fig. 4b–d).

In the miRNA inhibitor groups, the expression levels of *TYR*, *TYRP1*, and *TYRP2* mRNA in the transfected *miR*-*144a*-*3p* mimic, *miR*-*101a*-*3p*, and *miR*-*101a*-*3p* & *miR*-*144a*-*3p* (1:1) groups were significantly higher than those in the miRNA mimic group (*P *< 0.001, *P *< 0.001, and *P* < 0.001; Fig. 4b–d).

### miR-101a-3p and miR-144a-3p-modulated MITF and its downstream gene protein expression

**Fig. 5:**
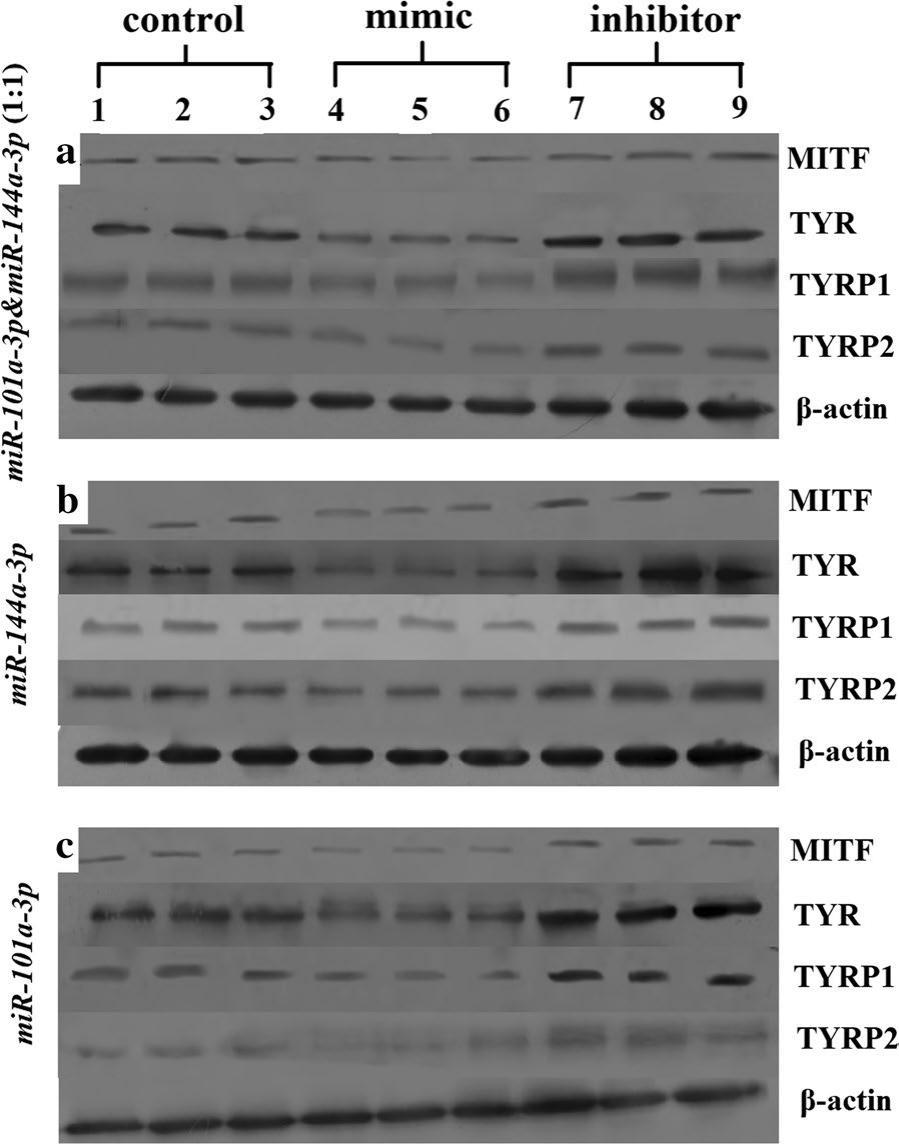
Results of the protein bands of MITF, TYR, TYRP1, and TYRP2 Western blot analysis in alpaca melanocytes. **a**–**c** represent the protein bands of MITF, TYR, TYRP1, and TYRP2 in *miR*-*101a*-*3p* & *miR*-*144a*-*3p* (1:1), *miR*-*144a*-*3p*, and *miR*-*101a*-*3p* groups, respectively. Lanes 1–3 show the control alpaca melanocytes transfected with nothing. Lanes 4–6 show the alpaca melanocytes transfected with mimic. Lanes 7–9 show alpaca melanocytes transfected with inhibitor. All samples are run in triplicate

**Fig. 6:**
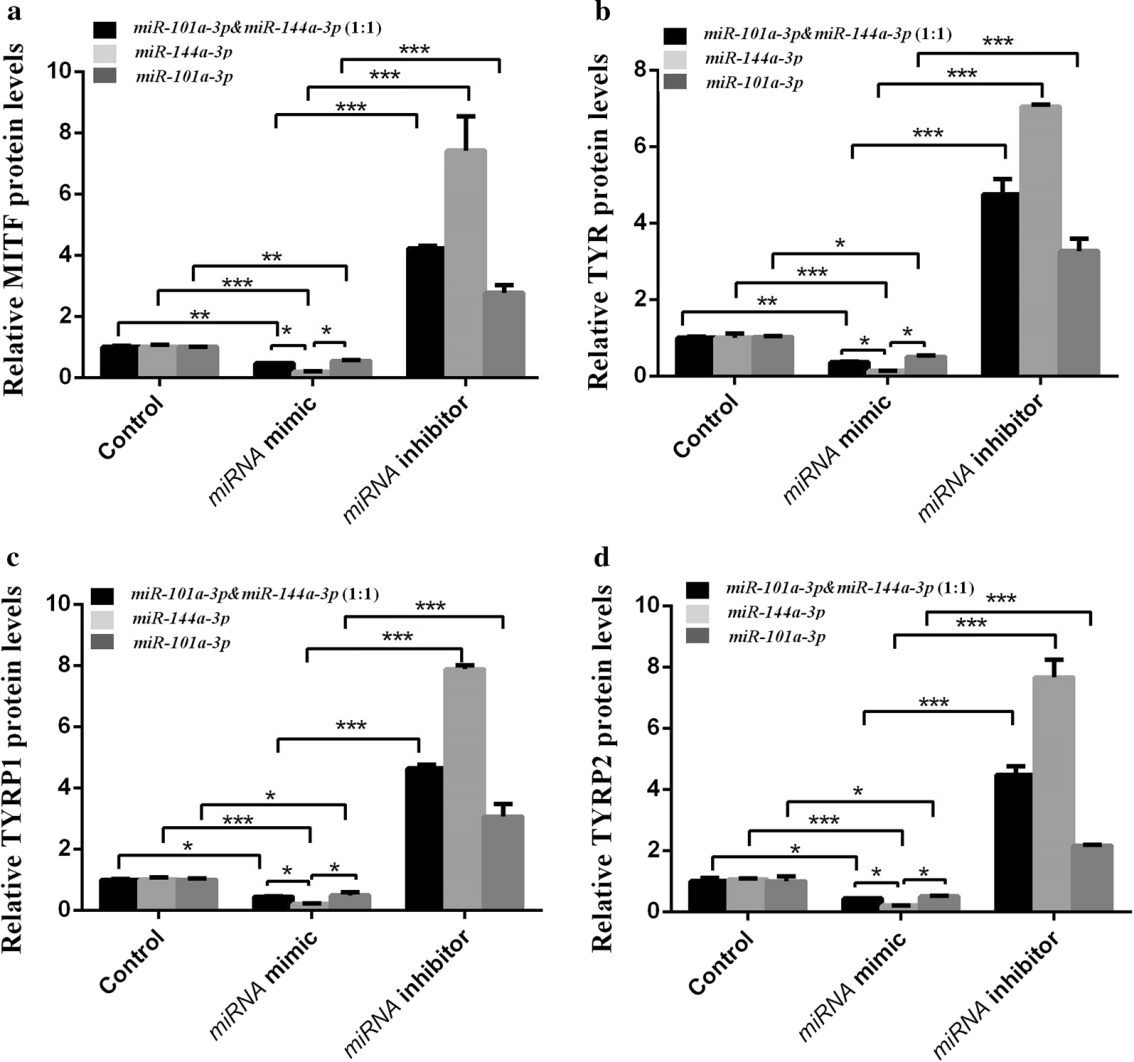
The protein expression of MITF, TYR, TYRP1 and TYRP2 in alpaca melanocytes. **a**–**d** represent the protein expression of MITF, TYR, TYRP1, and TYRP2, respectively. The control group represents the alpaca melanocytes normal cultured melanocytes. miRNA mimic group represents melanocytes transfected with miRNA mimic. The black column represents the alpaca melanocytes transfected with *miR*-*101a*-*3p* & *miR*-*144a*-*3p* (1:1) mimic, the light gray column represents the alpaca melanocytes transfected with *miR*-*144a*-*3p* mimic, and the dark gray column represents the alpaca melanocytes transfected with *miR*-*101a*-*3p* mimic. miRNA inhibitor group represents melanocytes transfected with miRNA inhibitor. The black column represents the alpaca melanocytes transfected with *miR*-*101a*-*3p* & *miR*-*144a*-*3p* (1:1) inhibitor, the light gray column represents the alpaca melanocytes transfected with *miR*-*144a*-*3p* inhibitor, and the dark gray represented alpaca melanocytes transfected with *miR*-*101a*-*3p* inhibitor (* means *P *< 0.05, ** means *P* < 0.01, *** means *P* < 0.001)

The MITF protein expression level of the transfected *miR*-*101a*-*3p* & *miR*-*144a*-*3p* (1:1) mimic, *miR*-*144a*-*3p* mimic, and *miR*-*101a*-*3p* mimic groups was significantly reduced compared with that of the control groups (*P* < 0.01, *P* < 0.001, and *P* < 0.01; Figs. 5, 6a). However, MITF expression in the inhibitor groups was significantly higher than that in the miRNA mimic groups (*P* < 0.001; Figs. 5, 6a). The TYR protein expression level of the transfected *miR*-*101a*-*3p* & *miR*-*144a*-*3p* (1:1) mimic, *miR*-*144a*-*3p* mimic, and *miR*-*101a*-*3p* mimic groups was significantly reduced compared with that of the control groups (*P* < 0.01, *P* < 0.001, and *P* < 0.05; Figs. 5, 6b). However, TYR expression in the inhibitor groups was significantly higher than that in the miRNA mimic groups (*P* < 0.001; Figs. 5, 6b). The TYRP1 and TYRP2 protein expression levels of the transfected *miR*-*101a*-*3p* & *miR*-*144a*-*3p* (1:1) mimic, *miR*-*144a*-*3p* mimic, *miR*-*101a*-*3p* mimic groups were significantly reduced compared with those of the control groups (*P* < 0.05, *P* < 0.001, and *P* < 0.05; Figs. 5, 6c, d, respectively). However, the TYRP1 and TYRP2 expression levels in the inhibitor groups were significantly higher than those in the miRNA mimic groups (*P* < 0.001; Figs. 5, 6c, d). In the miRNA mimic group, the MITF, TYR, TYRP1, and TYRP2 protein expression levels of the melanocytes transfected with *miR*-*144a*-*3p* mimic group were significantly lower than that of the melanocytes transfected with *miR*-*101a*-*3p* mimic and *miR*-*101a*-*3p* & *miR*-*144a*-*3p* (1:1) mimic groups (*P* < 0.05; Figs. 5, 6a–d).

### Protein localization and expression of miR-101-3p and miR-144-3p on MITF and its downstream gene

**Fig. 7:**
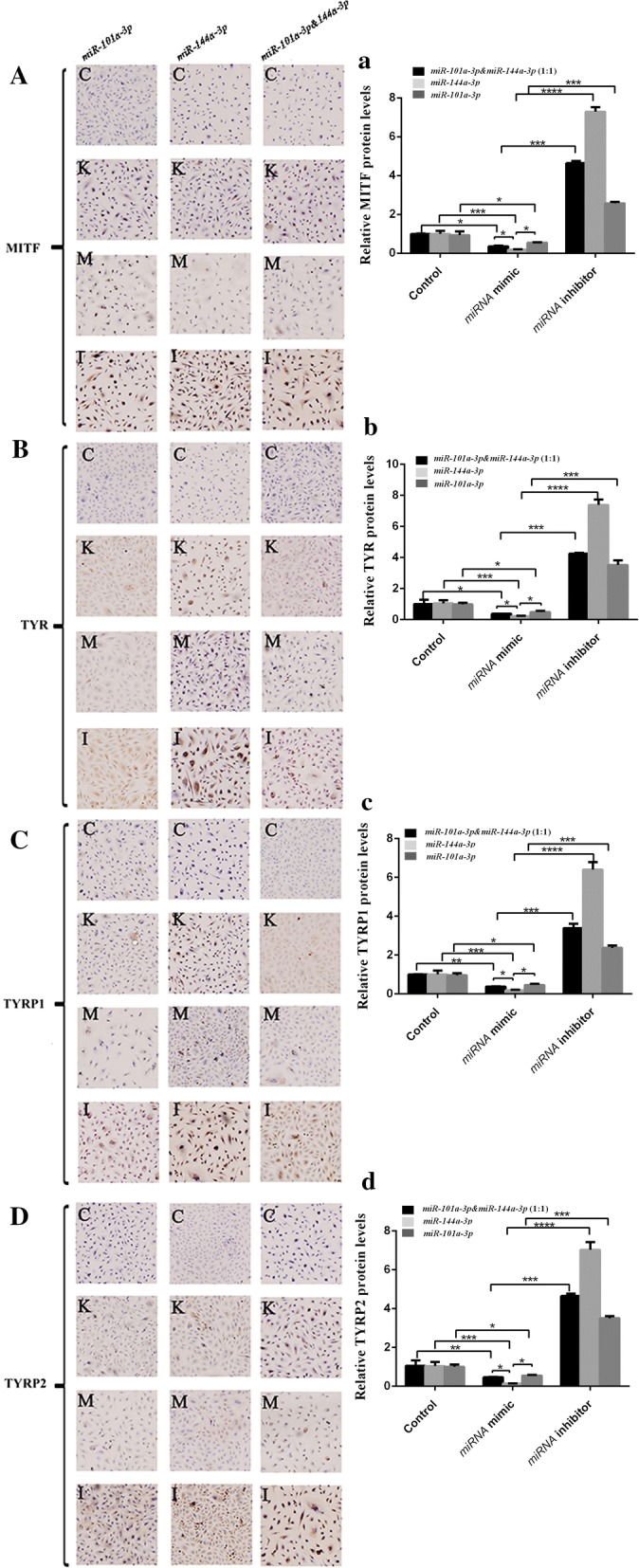
Immunohistochemically localization and analysis of MITF, TYR, TYRP1, TYRP2 protein in alpaca melanocyte. **c** represents the melanocytes without primary antibody, K represents the melanocytes with primary antibody, M represents mimic transfected group, and I represent inhibitor group. **A**–**D** represents the protein expression of MITF, TYR, TYRP1 and TYRP2, respectively. The black column represents *miR*-*101a*-*3p* & *miR*-*144a*-*3p* (1:1) group. The light gray column represents *miR*-*144a*-*3p* group. The dark gray column represents *miR*-*101a*-*3p* group (* means *P* < 0.05, ** means *P* < 0.01, *** means *P* < 0.001)

The immunohistochemistry images (Fig. 7A–D) show that MITF, TYR, TYRP1, and TYRP2 were expressed in the cytoplasm of melanocytes. Each protein expression was significantly reduced after transfection with *miR*-*101a*-*3p*, *miR*-*144a*-*3p*, and *miR*-*101a*-*3p* & *miR*-*144a*-*3p* (1:1) mimic. Optical density analysis of immunohistochemistry using Image-Pro Plus and the statistical analysis results of protein expression were consistent with the findings of Western blot analysis (Fig. 7A–D).

### Effects of miR-101-3p and miR-144-3p on melanin synthesis

**Fig. 8:**
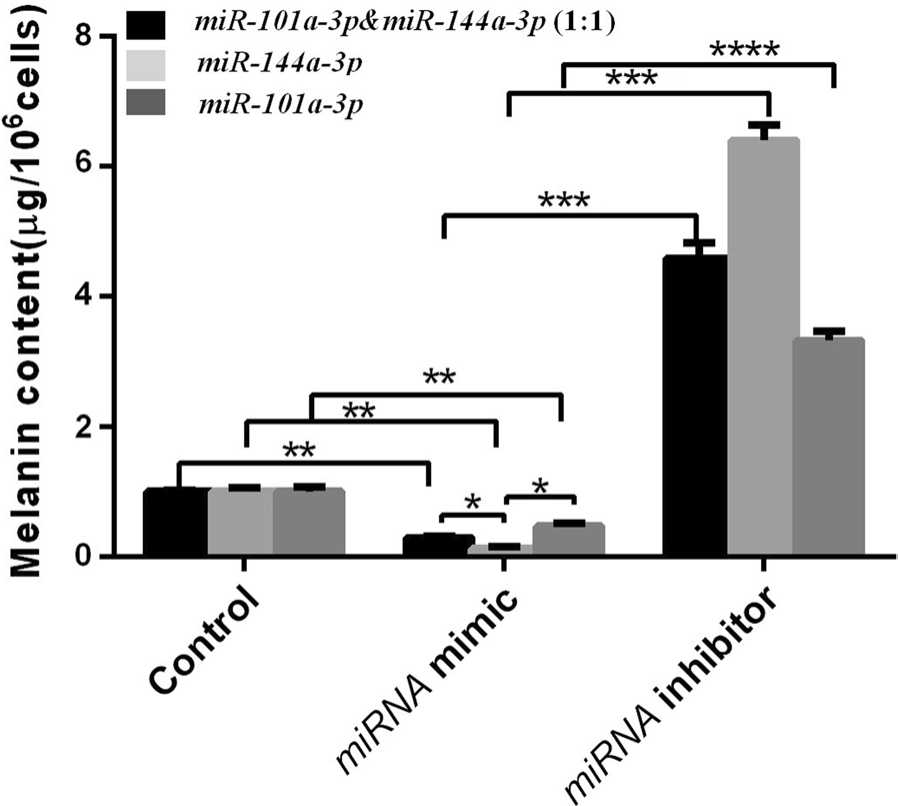
The relative production of melanin in melanocyte. The control group represents normal cultured melanocytes. The black column represents the melanocytes transfected with *miR*-*101a*-*3p* & *miR*-*144a*-*3p* (1:1) mimic group. The light gray column represents the melanocytes transfected with *miR*-*144a*-*3p* mimic group. The dark gray column represents the melanocytes transfected with *miR*-*101a*-*3p* mimic group. miRNA inhibitor group represents melanocytes transfected with miRNA inhibitor. The black column represents the alpaca melanocytes transfected with *miR*-*101a*-*3p* & *miR*-*144a*-*3p* (1:1) inhibitor, the light gray column represents the alpaca melanocytes transfected with *miR*-*144a*-*3p* inhibitor, and the dark gray represents the alpaca melanocytes transfected with *miR*-*101a*-*3p* inhibitor (* means *P *< 0.05, ** means *P* < 0.01, *** means *P *< 0.001)

Compared among the melanin content of the control, mimic and inhibitor groups, the melanin content of the *miR*-*101a*-*3p* & *miR*-*144a*-*3p* (1:1) mimic, *miR*-*144a*-*3p* mimic, and *miR*-*101a*-*3p* mimic groups significantly decreased (control group: *P *< 0.01; inhibitor group: *P *< 0.001; Fig. 8), and the *miR*-*144a*-*3p* mimic group was the lowest (*P *< 0.05; Fig. 8). The *miR*-*101a*-*3p* & *miR*-*144a*-*3p* (1:1) mimic group was lower than the *miR*-*101a*-*3p* mimic group (*P* < 0.05; Fig. 8).

## Discussion

Melanin is synthesized by melanocytes, and melanin deposited in hair determines its color. MITF regulates many pigment-related enzymes and is a critical transcription factor. *TYR*, *TYRP1*, and *TYRP2* are important downstream genes of MITF and key enzymes for pigmentation. Therefore, many different external signals affect melanin production by regulating the expression of *MITF* and its downstream genes [19, 20]. miRNAs are highly conserved non-coding RNAs that inhibit post-transcriptional regulation of target genes by binding to the target genes.

*miR*-*203* was first identified miRNA in skin, which is involved in the regulation of melanocyte pigmentation [21]. The expression level of *miR*-*203* is increased in the skin of patients with psoriasis compared with that in normal skin, and *miR*-*203* plays an important role in mouse epidermal differentiation [22, 23]. *miR*-*137* acts on MITF by binding to the specific bases of *MITF* mRNA and affects the expression of its downstream genes *TYR*, *TYRP1*, and *TYRP2*, thereby regulating pigmentation [24]. The role of *miR*-*340* and *miR*-*25* in the formation of coat color has been reported in the literature [25, 26]. *miR*-*101a*-*3p* and *miR*-*144a*-*3p* play important roles in cell apoptosis, immunity, and tumor suppression [27, 28]. However, the *miR*-*101*-*3p* and *miR*-*144*-*3p* hair color pigmentation mechanisms remain largely unknown. Only one study has reported that *miR*-*101*-*3p* inhibits melanoma proliferation and expansion by regulating MITF in melanoma [29].

miRNA regulation of target genes is at the post-transcriptional level, and cleavage is the mechanism of target gene mRNA; its translational inhibition depends mainly on its degree of complementation with the target gene mRNA sequence [30]. In our study, software predictions and analysis demonstrated that *miR*-*101a*-*3p* and *miR*-*144a*-*3p* targeted the same site of *MITF* 3′UTR, and the degree of *miR*-*144a*-*3p* regulating *MITF* was stronger than that of *miR*-*101a*-*3p*. After the overexpression of *miR*-*101a*-*3p* and *miR*-*144a*-*3p*, the expression level of *MITF* mRNA remained the same, and the MITF protein expression decreased. The mRNA and protein expression levels of TYR, TYRP1, and TYRP2 significantly decreased, and the melanin content also decreased. The regulation of *miR*-*144a*-*3p* on the expression of MITF protein was stronger than that of *miR*-*101*-*3p*, and the melanin content was lower than the *miR*-*101a*-*3p* group. The two miRNAs regulated the expression of *MITF*, which further affected downstream genes (*TYR, TYRP1*, and *TYRP2*) to fulfill the regulation of melanocyte pigmentation.

## Conclusion

*miR*-*101*-*3p* and *miR*-*144a*-*3p* exhibited the same binding site on the 3′UTR of *MITF*. The inhibitory effect of *miR*-*144a*-*3p* was stronger than that of *miR*-*101*-*3p* in alpaca melanocytes, which decreased melanin production.

## Data Availability

The datasets supporting the findings and all data generated or analyzed during this study are included in this article.

## Abbreviations

MITF: microphthalmia-associated transcription factor
TYR: tyrosinase
TYRP1: tyrosinase-related proteins 1
TYRP2: tyrosinase-related proteins 2
